# Diabetes Burden and Healthcare Barriers Among Cataract Patients in Imo State, Nigeria

**DOI:** 10.5334/aogh.5168

**Published:** 2026-09-07

**Authors:** Kamsi Oparaugo, Parker Cox, Andrey Kharlamov, Kelechi Mezu-Nnabue, Udo Ubani, Olachi J. Mezu-Ndubuisi

**Affiliations:** 1 University of Rochester, Rochester, NY, USA; 2 Mezu International Foundation, Pikesville, MD, USA; 3 Abia State University, Uturu, Abia State, Nigeria

**Keywords:** cataract, diabetes, diabetes burden score, barriers to care, socio-economic status, drinking water, comorbidities

## Abstract

*Background:* The coexistence of diabetes and cataracts increases the risk of poor surgical outcomes. Cataract patients with uncontrolled diabetes and other comorbidities were unsafe for same-day surgery, which excluded them from free cataract surgeries during a medical mission outreach in rural Imo State, Nigeria.

*Objective:* This study aims to identify barriers to diabetes control among cataract patients in the community, to improve health outcomes and surgical readiness in short-term medical missions.

*Methods:* A total of 45 cataract patients aged 40–94 years received point-of-care testing for fasting blood sugar, intraocular pressure (IOP), and hemoglobin levels. Structured questionnaires assessed socio-economic status (SES), diabetes burden, and barriers to care.

*Findings:* Despite reported adherence to daily medications (p = 0.0007), many patients had high diabetes burden scores, which were significantly associated with frequent symptoms (p < 0.00003), and more chronic diseases (p < 0.008). Elevated IOP, a glaucoma risk factor, was linked to private drinking-water sources (p = 0.008), and herb use was associated with lower hematocrit (p = 0.040), suggesting anemia risk. Lower hematocrit further correlated with more advanced cataracts (p = 0.018). High barriers-to-care scores were associated with the absence of a caregiver (p = 0.005), limited food choices (p < 0.001), the frequency of medical checks (p < 0.001), and a patient’s medication source (p = 0.030). Patients who struggled with transportation barriers reported needing help with daily tasks like going to the market or cooking meals (p = 0.004).

*Conclusion:* Disease severity in cataract patients is influenced by overlapping medical, financial, and social barriers. Providing tools for effective diabetes self-monitoring and mitigating comorbidities through community-tailored interventions can improve health stability and future surgical access in this rural community.

## Introduction

Global health initiatives to provide short-term aid to resource-limited communities can be challenging due to limited timelines. The impact of these initiatives on long-term well-being is dependent upon risk factor assessments and health impact education [1]. Preventable blindness remains a significant burden in low-income countries, with cataracts being a major cause of preventable blindness worldwide, especially in Imo State, Nigeria [2, 3]. The Mezu International Foundation (MIF) is a nonprofit organization that conducts annual medical and humanitarian missions in Emekuku, a rural community in Owerri, Imo State, South-East Nigeria. During a free cataract surgery outreach targeting 50 cataract patients, only 10 qualified for surgery due to severe untreated medical comorbidities that posed safety threats for same-day surgery in an outpatient facility, such as diabetes, hypertension, glaucoma, and anemia [1]. A better understanding of the barriers to care of comorbidities in cataract patients is needed.

Diabetes is a systemic disease that is often associated with cataract formation and worsens cataract progression, placing patients at an increased risk for poor surgery outcomes [4]. In rural communities with limited resources, cataracts often remain uncorrected and lead to blindness [5]. While cataract surgery is more accessible in developed countries, the co-existence of diabetes and cataracts poses a global threat to cataract patients in low-, middle-, and high-income countries [6]. According to the World Health Organization, diabetes prevalence has dramatically risen across countries of varied incomes. 38.4 million Americans are plagued by diabetes as of 2021 [7, 8]. Despite this, the global diabetes prevalence is expected to surpass a billion, increasing the burden of comorbid diabetes among cataract patients worldwide [9]. In Nigeria, the prevalence of type 2 diabetes had risen from 2.0% in 1990 to 5.77% in 2015 and affects 4.6% of individuals living in the southeastern region of the country, including Imo State, with a large portion of patients remaining undiagnosed [10, 11].

Since successful diabetes therapy relies heavily on self-management, a common barrier to treatment across both middle- and low-income individuals is the lack of patient-tailored care [12]. These patients are at a particularly higher risk of complications, including hyper and hypoglycemia, under- and over-nutrition, and diabetic retinopathy, compared to those in developed nations. Hyperglycemia is highly prevalent among type 2 diabetic patients, with approximately 40%–68% of patients unable to maintain a hemoglobin A1c less than 7% [13]. Nigeria-specific data surrounding hypoglycemia are limited, primarily due to insufficient data collection; however, the reported prevalence of hypoglycemia among type 2 diabetic patients in other surrounding sub-Saharan nations ranges from 13.1% to 41.9% [14–16]. Black Africans have a twofold increased risk compared to other groups [17]. The wide use of sulfonylureas due to cost and availability increases this risk of hypoglycemia, particularly in settings where patients have inconsistent food intake or limited access to blood glucose monitoring. Dietary patterns further complicate diabetes management in rural settings. Many patients rely on staple foods such as yam, rice, and fried foods, which are often high in carbohydrates. Food insecurity, combined with limited access to diverse or protein-rich foods, can lead to both undernutrition and poor glycemic control. In addition, physical limitations and transportation barriers may further restrict access to appropriate foods, thus reinforcing the cycle of high diabetes burden and poor disease management. Diabetes patients in rural low-income countries are plagued with a higher risk of complications and several barriers to achieving adequate control of their chronic medical conditions.

The objective of this study is to determine the socio-economic and systemic barriers to care among cataract patients in a rural community of South-East Nigeria. This would better inform health professionals and public health missions on patient-tailored ways to optimize care of cataract patients with comorbidities.

## Methods

This was a cross-sectional study conducted at the Mezu International Foundation Medical Center, in a rural community in Imo State, Nigeria, during a free annual medical mission. The study was performed in accordance with the principles of the Declaration of Helsinki, and Institutional Review Board approval was obtained from Abia State University, Uturu, Abia State, Nigeria (ABSU/REC/OPT/001/2024).

Patients were eligible for the study if they had a confirmed diagnosis of cataracts, regardless of severity. Selected patients were men and women aged 40 to 94 years old. Informed consent of all adults was obtained before participation.

Patients underwent point-of-care testing of blood glucose, intraocular pressure (IOP), hemoglobin (Hgb), and hematocrit (HCT) to test for the presence of comorbidities. Most diabetes cases were consistent with type 2 diabetes based on the age and measured fasting blood glucose levels. Fasting blood glucose levels, measured in mg/dL via point-of-care testing, were used as an indicator of diabetes risk in the absence of HbA1c, which was not available in this low-resource setting. Previously diagnosed diabetes patients were asked to self-report their oral diabetes medications, as well as insulin injection use where applicable, and data on prescribed medication frequency, access to medications, and use of non-prescribed treatments, including herbal remedies, were also collected. Comorbid conditions, including both systemic and ocular diseases, were recorded in Table 4 to describe the clinical profile of the study population. Cataract severity was graded as 1+, 2+, or 3+ nuclear sclerosis or cortical cataracts by the optometrist. IOP and blood glucose were treated as continuous variables for Spearman associations, with glaucoma risk defined by elevated IOP values >21 mmHg. Anemia was defined as Hgb <12.0 g/dL. Hypertension was defined by systolic pressure ≥120 mmHg or diastolic pressure ≥80 mmHg.

Questionnaires administered to eligible patients include a modified MIF socio-economic status (SES) questionnaire [18], along with new questionnaires developed to assess diabetes burden and barriers to care. SES was assessed using indicators such as education, housing type, and ownership of electronics. Diabetes burden was assessed using indicators of diabetes risk, diabetes management history, treatment adherence, and diabetes severity. Barriers to care evaluated variables such as financial difficulty, caregiver presence, needing help with daily tasks, smoking, clinic/pharmacy accessibility, soda and sweets consumption, herb reliance, frequency of glucose checks, exercise frequency, delayed last doctor visit, and medication source.

## Statistical Analysis

All statistical analyses were conducted using R (version 4.6.1; R Core Team), RStudio, and GraphPad Prism (version 10). Continuous numeric variables are presented as mean ± SD, or median with interquartile range (IQR), where appropriate. Because our study variables were not normally distributed and the sample size was limited, several non-parametric statistical tests were used.

Spearman rank correlations were used to evaluate associations between continuous or ordinal variables. For adjusted correlation analyses, partial Spearman correlations were calculated in R by ranking variables and applying Pearson’s correlation to the ranked data using the ppcor package. This allowed us to examine the relationships between variables while adjusting for selected covariates.

Group differences in diabetes burden score were assessed using the Mann-Whitney U test since the score is not normally distributed, making a rank-based comparison more appropriate than a parametric test.

Associations between categorical variables were assessed using Fisher’s exact test, which calculates an exact probability of association and is more appropriate than the chi-square test when expected cell counts are small, which was the case in this sample. Specifically, Fisher’s exact test was used to examine whether medication adherence differed by drinking-water source group.

We fitted linear regression models to examine the association between each barrier-to-care variable and diabetes burden score. Each model included one barrier-to-care variable as the primary predictor and adjusted for three social determinants of health (SDOH) as potential confounders: type of home, main source of drinking water, and main source of transportation. A P-value < .05 was considered statistically significant.

## Results

### Age was positively associated with chronic medical illness

A cross-sectional analysis was completed on 45 patients from 40 to 94 years old. There were 29 females, 13 males, and 3 who did not indicate their gender, but there were no statistically significant associations between sex and variables of interest. Mean age was 64.53 ± 12.70 (n = 43).

Older patients were more likely to reside in privately owned cement homes (r = 0.40; 95% CI, 0.11–0.63, p = 0.007), have a caregiver present in the household (r = 0.42, p = 0.005), and report a higher number of chronic medical conditions (r = 0.41, p = 0.008) compared to younger patients. The average systolic blood pressure (SBP) was noted to be 140.5 ± 30.12 mmHg (n = 32).

### Socio-Economic status influenced diet, access to improved home infrastructures, and sources of drinking-water

Participants with a higher SES scores were more likely to own personal electronics (p < 0.0001, r = 0.84), consume animal protein (chicken, meat, or fish) more frequently (p = 0.0295), and more likely to have a personal borehole (private) source of drinking water (p < 0.0002), while those with lower SES scores consume chicken or meat only a few times per month (p = 0.0295), were more likely to receive food from charity sources (p = 0.007), have limited transportation (p = 0.012), and were less likely to live in privately owned homes (p = 0.026). These patients were instead more likely to live in more crowded sleeping arrangements, where three or more share a bed, as graded in Table 1.

**Table 1:** Modified MIF socio-economic status (SES) questionnaire. This SES questionnaire is structured to use nine brief, context-specific questions to further classify patients into low, middle, or high socio-economic groups within the rural Nigerian communities of this study’s focus. The aim of the questionnaire is to assess multiple domains that reflect various aspects of daily living conditions among these individuals.

MODIFIED MIF SOCIO-ECONOMIC STATUS (SES) QUESTIONNAIRE	
**Question**	**Response Options (Score)**
1. Highest level of education attained by yourself or any of your parents:	University (4) Post-secondary education/technical college/polytechnic (3) Secondary education (2) Primary education (1) Pre-primary education (0) Never enrolled in school (–1)
2. What type of home do you live in?	Own cement home (with stairs) (4) Own cement home (bungalow) (3) Own thatched/mud home (2) Rent a home or apartment (1) Shared family home (apartment/flat) (0) Homeless (shelter/open streets) (–1)
3. What personal electronics do you own? (1 point for each electronic owned)	Radio (1) TV (1) Cell phone (1) Generator (1) Cable or Satellite (1)
4. Sleeping arrangements (number of people per bed):	1 per bed (4) 2 per bed (3) 3 per bed (2) 1–3 per mat/mattress on the floor/hard floor (1) >3 people per bed (0)
5. Main source of drinking water (score highest option given):	Vendor, bottled water, or personal bore hole (4) Pipe, tap water, or shared bore hole (3) Well water (2) River, lake, or stream (1) Rainwater (0)
6. What type of toilet facilities does your household use?	Own toilet (manual/self-flushing) (4) Own toilet (flush with bucket) (3) Shared toilet (<3 families) (2) Own pit/latrine (1) Public facilities (0) None/bush (–1)
7. Do you receive food from the following places?	Do you eat in a restaurant once or more a week? (4) Do you shop at a supermarket once a week or more? (3) None of the below (2) Charity or food program (1) Street aid and/or begging (0)
8. How many times do you and/or your family eat chicken, meat or fish?	Every day (4) More than 3 times a week (3) Less than 3 times a week (2) Once a week (1) A few times a month (0)
9. What is your main source of transportation?	By car (with air conditioning) (4) By car (no air conditioning) (3) On motorcycle (2) On bicycle (1) Public transport (0) On foot (–1)
**Socio-economic status (SES) score: −4 to 11 Low; 12 to 23 Middle; 24 to 36 High**	

Owning more personal electronics, a marker for higher SES, was also associated with better toilet facilities at home (p < 0.00001, r = 0.70) and access to a personal borehole for drinking water (p = 0.002, r = 0.46). While patients who owned fewer electronics were less likely to obtain food from a restaurant or supermarket (r = 0.40, p = 0.007) and more likely to report barriers to clinic or pharmacy access (r = 0.40, p = 0.007).

### Patients with improved home infrastructures reported less barriers to diabetes care and more medication consumption

**Figure 1:**
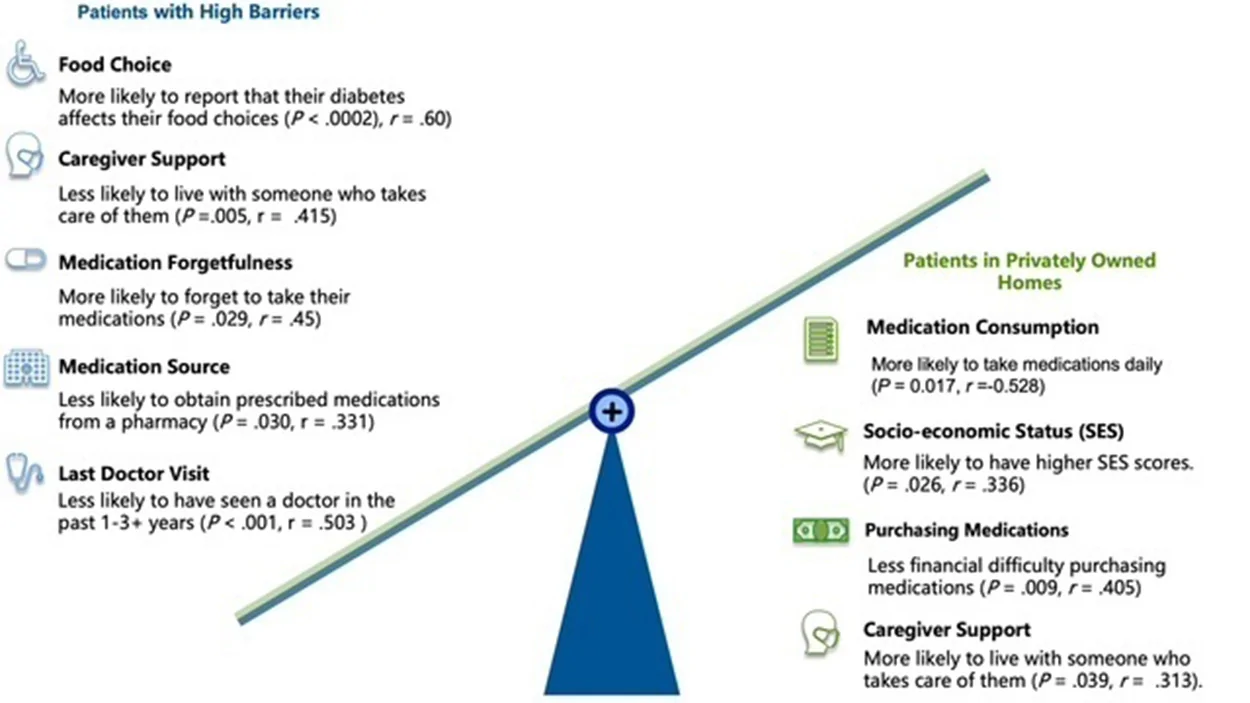
Direct associations with barriers to diabetes treatment scores. This figure represents an infographic of significant direct associations between barriers to care and key socio-economic factors among cataract patients. Patients with more barriers often faced financial difficulty and limited access to health needs, while those in private homes were associated with fewer barriers to care scores, and frequent medication consumption. Note: Significance denotes P values < 0.05.

Living in privately owned cement homes was positively associated with higher SES scores (r = 0.34, p = 0.026). Patients in privately owned cement homes were less likely to experience financial difficulty buying diabetes medications (r = 0.41, p = 0.009) and more likely to have a live-in caregiver at home (r = 0.31, p = 0.039). A moderate association was found between living in privately owned cement housing and lower barriers-to-care scores (r = 0.33, p = 0.030), depicted through Figure 1. Additionally, patients in privately owned homes were more likely to report taking medications/injections daily (r = −0.528, p = 0.017, n = 20) when asked “How often do you take your medications/injections?” in Table 2.

**Table 2:** Diabetes burden questionnaire. The diabetes burden questionnaire depicts a six-question system to classify patients’ diabetes severity or risk as Low, Moderate, or High based on treatment adherence, prescribed therapy, and symptom burden.

DIABETES BURDEN QUESTIONNAIRE	
**Question**	**Response Options (Score)**
1. Which of these are you prescribed for your diabetes?	Injections (1) Tablets (1) Both injections and tablets (−1) None (0)
2. How often are you supposed to take your medications according to your doctor?	My doctor has not prescribed any diabetes medication (1) Only on some days per week (1) Once a week or monthly (0) Daily, after every meal (–1)
3. How often do you take your medications/injections?	Once a week or monthly (1) Few times a week (0) Daily or after every meal (–1)
4. Do you have any symptoms due to your diabetes? Mark all that apply.	None (1) Tingling/numbness of legs and fingers (0) Blurry vision or dizziness (–1) Ulcers or non-healing wounds (–1)
5. How often do your symptoms occur?	Few times a week (1) Once a week or monthly (0) Daily, after every meal (–1)
6. Does your diabetes affect your choice of food?	No (0) Yes (–1)
**Diabetes Burden Score: −6 to –3 High; −2 to 1 Moderate; 2 to 5 Low**	

### Lower SES diabetes patients struggle with food insecurity, transportation barriers, and daily essential tasks

Patients receiving food from charity sources were more likely to be older (r = −0.88, p = 0.019, n = 7), own fewer electronics (r = 0.40, p = 0.007), have lower SES (r = 0.40, p = 0.007), and report greater physical mobility barriers when traveling to clinics or pharmacies (r = 0.36, p = 0.019), detailed in Table 3.

**Table 3:** Barriers-to-care questionnaire. The barriers-to-care questionnaire aims to measure the impact of various practical, cultural, and health system barriers and subsequent interference with self-management of diabetes among patients within rural Nigeria.

BARRIERS-TO-CARE QUESTIONNAIRE	
**Question**	**Response Options (Score)**
1. Do you have financial difficulty purchasing your diabetes medications?	No (1) Sometimes (0) Yes (−1)
2. Are there people who live with you and take care of you at home?	Yes (1) Sometimes (0) No (−1)
3. Do you need help to cook, clean, farm, or go to the market on your own?	No (1) Sometimes (0) Yes (−1)
4. Do you smoke?	No (1) I do not, but a family member I live with does (0) Yes (−1)
5. What barriers do you encounter when traveling to a clinic/pharmacy?	Do not have any issues walking or driving to the pharmacy (1) Can walk, but live too far from the closest clinic/pharmacy (0) Use a wheelchair or walking stick and no car (−1)
6. How many times did you drink soda or take sweets in the past week?	<1 time per week (0) Less than 3–4 times per week (1) ≥3–4 times per week (−1)
7. Do you take any herbs/natural remedies to manage your diabetes?	Yes, specify (1) No (0)
8. How often do you forget to take your diabetes medications?	Never or once in a day (1) 2 or more times a week (0) Stopped taking them (−1)
9. How often do you check your blood glucose levels?	≥1 time per week (1) Monthly (0) <1 time per week (−1)
10. How often do you exercise (e.g., bicycle riding, farming, jogging, dancing)?	Every day (1) About 3–4 times per week (0) <2 times per week (−1)
11. What is the average range of your blood glucose levels?	Open response (no score)
12. When was your last visit to a doctor?	<2 months (4) 3–6 months (3) 6–12 months (2) 1–3 years (1) >3 years (0)
13. Where do you obtain medication?	From a pharmacy/chemist with a prescription (3) From a pharmacy/chemist without a prescription (2) From a friend or relative (1) Other, specify (0)
**Barriers-to-care score: −9 to 0 High; 1 to 10 Moderate; 11 to 17 Low**	

Patients with limited transportation options (walking or public transport) were more likely to have lower SES scores (r = 0.37, p = 0.012) and more likely to report needing help with daily activities such as cooking, cleaning, farming, or going to the market on their own. (r = 0.42, p = 0.004).

### SES scores were significantly associated with multiple indicators of education, housing stability, and access to essential resources

Participants with higher SES were more likely to have attained higher levels of education (r = 0.57, p < 0.001), live in privately owned homes (r = 0.34, p = 0.026), and own more personal electronics (r = 0.84, p < 0.0001). SES was also strongly correlated with access to improved home infrastructure, as participants with higher SES were more likely to use private drinking water sources (r = 0.53, p < 0.0002) and modern toilet facilities (r = 0.76, p < 0.0001). Food access and an animal protein diet were also linked to SES. Patients with higher SES consumed chicken, meat, or fish more frequently (r = 0.33, p = 0.030) and were more likely to obtain food from restaurants or supermarkets rather than charitable sources (r = 0.40, p = 0.007).

### Barriers-to-care score correlated with socio-economic factors and health-related behaviors

Patients who reported that their diabetes affects their food choices were more likely to experience difficulty accessing a clinic/pharmacy due to physical disability, yet still had no car or lived too far from the nearest clinic (r = 0.39, p = 0.025). Patients limited by food choices were less likely to have visited a doctor recently (r = 0.40, p < 0.01), and more likely to have higher barriers-to-care scores (r = 0.60, p < 0.0002; Figure 1). There was a strong association between financial difficulty and significantly high barriers-to-care scores (r = 0.62, p < 0.00002). Patients who reported financial difficulty purchasing medications were more likely to experience several other compounding barriers. They were less likely to have caregivers at home (r = 0.49, p = 0.001), more likely to need help with daily tasks like cooking, cleaning, or going to the market (r = 0.38, p = 0.015), and more likely to have lower Hgb (r = 0.50, p = 0.002) and lower HCT levels. On the other hand, patients who had caregiver support (r = 0.42, p = 0.005) were less likely to have high barriers-to-care scores (p = 0.005, r = 0.415), and less likely to have low Hgb levels (r = 0.46, p = 0.003).

High barriers-to-care scores, derived through Table 3, were also correlated with forgetting medications (r = 0.45, p = 0.029) and failing to check blood glucose levels frequently (r = 0.54, p < 0.001). The most frequent check seen in all patients was weekly. No patient checked their glucose levels daily.

More frequent (weekly) glucose checks were associated with recent doctor visits (r = 0.42, p = 0.010), and a lower chance of elevated IOP (r = −0.59, p = 0.023). Patients who experienced high barriers to care were less likely to have seen a doctor in the past 1–3+ years (p < 0.001, r = 0.503) and less likely to receive their medications from a pharmacy *with a prescription* (p = 0.030, r = 0.331), but rather relied on alternatives from an herbalist or non-prescribed medication from a pharmacy.

### Health-Seeking behaviors and patients’ attempts to reduce their diabetes symptoms may be related to increased anemia risks

Patients who obtained their medications from these alternative sources were more likely to exercise more frequently than the patients with prescriptions (r = −0.39, p = 0.014). This may partly be due to the survey criteria including farming activity as exercise, alongside bicycle riding, jogging, or dancing, as seen in Table 3.

Patients who reported using herbs or natural remedies were less likely to consume sugary foods and sodas multiple times per week (r = 0.38, p = 0.020, n = 38). However, herb users were more likely to have lower HCT levels (r = −0.47, p = 0.040, n = 19).

There was a strong correlation between herb use and living in more crowded sleeping arrangements where 3 or more share a bed (r = −0.41, p = 0.007, n = 42), possibly indicating socio-economic constraints that shape both living conditions and health behaviors.

### Requiring help with daily tasks was associated with anemia risk and advanced cataracts

**Figure 2:**
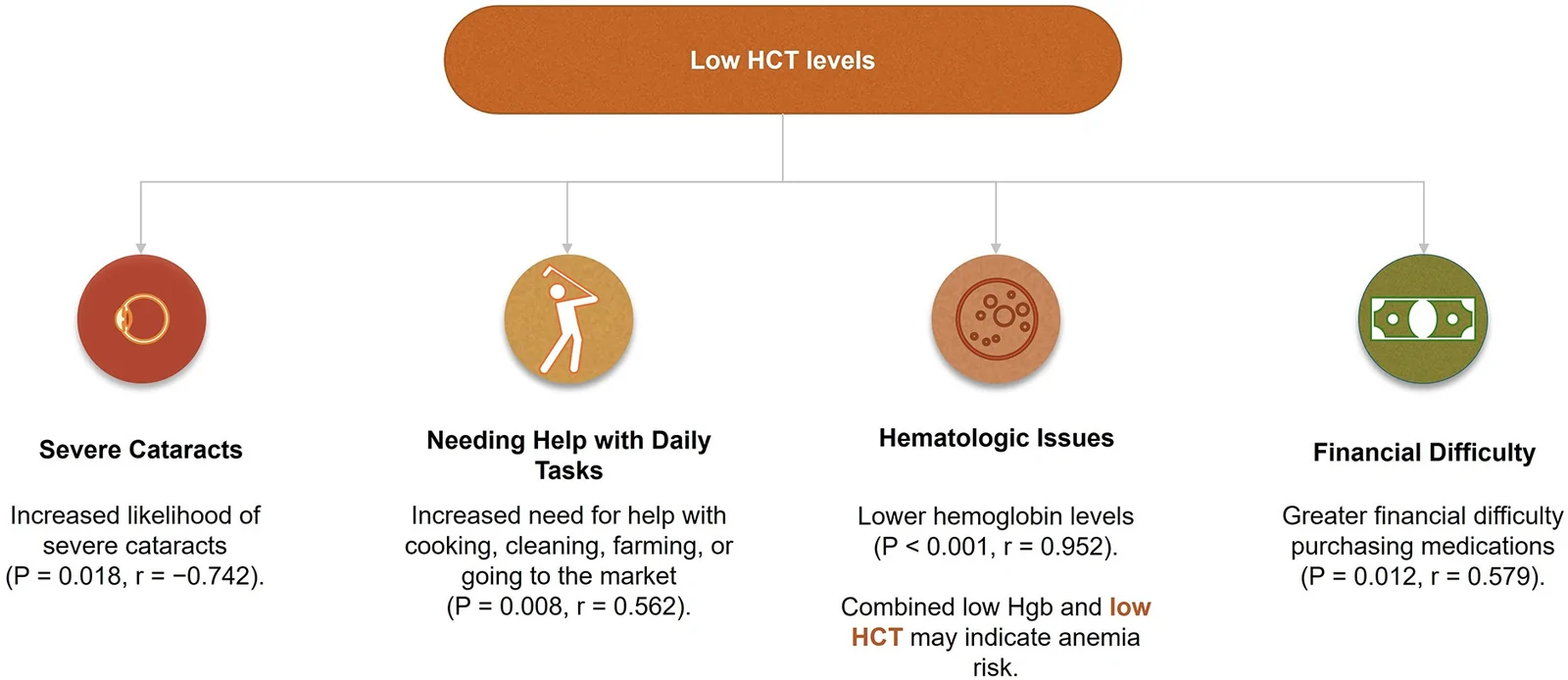
Low hematocrit (HCT) levels and associated factors. In this figure, an infographic summarizing key factors associated with lower HCT levels is shown. There was an association between low HCT and herb use (p = 0.041). Patients with low HCT were also more likely to have severe cataracts (p = 0.018), need help with daily tasks (p = 0.008), have lower hemoglobin levels (p < 0.001), and experience greater financial difficulty purchasing medications (p = 0.012). These findings suggest that anemia risk may be compounded by both clinical, behavioral, and socio-economic challenges in this population. Note: HCT denotes Hematocrit

Patients who reported needing help with daily tasks, such as cleaning, cooking, farming/going to the market, were more likely to rely on public transportation or walking as their primary transportation source. They were also more likely to have lower Hgb (r = 0.36, p = 0.023) and lower HCT levels (r = 0.56, p = 0.008). Patients who reported needing more help were more likely to have visited a doctor recently (r = −0.35, p = 0.022), yet more likely to have advanced cataracts (r = −0.45, p = 0.030) graded in Table 4. Low HCT was also further associated with low Hgb (r = 0.95, p < 0.001) and more advanced cataracts (r = −0.74, p = 0.018), as illustrated in Figure 2.

**Table 4:** Post-assessment clinical information. The MIF original post-assessment clinical information survey was used by clinicians to document systemic comorbidities and corresponding severity among patients within the study in a single standardized format to allow for comparison.

POST-ASSESSMENT CLINICAL INFORMATION		
SECTION	ITEM	DESCRIPTION/OPTIONS
Medications	Current medications being taken	Open response
Laboratory/vitals	Hemoglobin (Hgb)	______ mg/dL
	Blood pressure	______ mmHg
	Blood glucose	______ mg/dL
	Intraocular pressure (IOP) OD	______ mmHg
	Intraocular pressure (IOP) OS	______ mmHg
Other findings	Other	Open response
Cataracts staging	Cataracts OD	______ (mild: trace, 1+, 1–2+; mature: 3+ and higher; coding for dataset: mild = 0, mature = 1)
	Cataracts OS	______ (mild: trace, 1+, 1–2+; mature: 3+ and higher; coding for dataset: mild = 0, mature = 1)
Medical diagnosis (check all that apply)	Diabetes	
	Anemia	
	Cataracts	
	Glaucoma	
	Hypertension	
	Other	______________________________

### Private drinking water sources, a variable for higher SES, was associated with glaucoma risk

**Figure 3:**
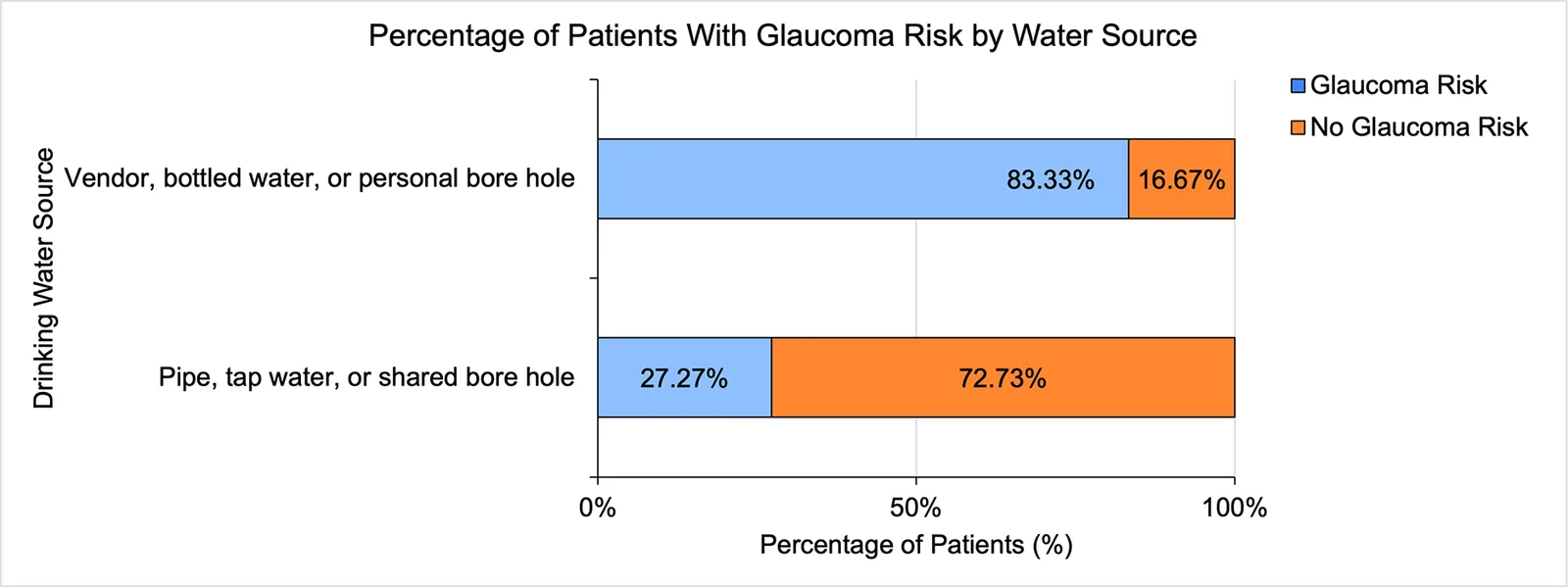
Drinking water source and risk for glaucoma. This figure depicts the association between drinking water source and the risk for glaucoma. Each bar shows the percentage of patients within a drinking-water source category who are at risk for glaucoma, IOP greater than 21 mmHg in either eye. Patients using “Vendor, Bottled, or Personal Bore Hole” water had the highest glaucoma risk (83.33%), while Pipe/Tap or Shared Bore Hole users had the lowest observed glaucoma risk (27.27%). Bars are grouped by drinking-water source, with glaucoma risk status indicated by color (blue for risk and orange for no risk). Note: IOP denotes intraocular pressure. Glaucoma risk is defined by IOP > 21 mmHg, while No Glaucoma risk is defined by IOP ≤ 21 mmHg.

Table 5 shows that drinking vendor/bottled water or having access to a personal borehole (private sources) was associated with owning more personal electronics (r = 0.460, p = 0.002), better toilet facilities (p = 0.006, r = 0.406), higher SES scores (r = 0.532, p < 0.0002), yet higher IOP among patients (r = 0.51, p = 0.015; Figure 3). There were also strong positive correlations between a patient’s main source of drinking water and elevated IOP in both the right eye (r = 0.49, p = 0.018) and left eye (r = 0.55, p = 0.008).

**Table 5:** Drinking water source correlations. This table examined correlations between drinking water source and any other significantly associated variables. Water source was scored ordinally, where higher scores indicate a private water source (with a score of 3 = pipe/tap/shared borehole; while 4 = vendor/bottled/personal borehole). The highest IOP’s 95% CI for Spearman’s r was computed using the DescTools package in R. Sample size (n) varied across the rows due to missing data for some individual variables. A p-value of less than 0.05 was considered statistically significant. Highest IOP was defined as the greater value between IOP measured in the right eye (OD) and left eye (OS).

DRINKING WATER SOURCE CORRELATIONS					
EXPOSURE	ASSOCIATED VARIABLE	N	SPEARMAN R	95% CI	P-VALUE
Private sources of drinking water	Higher SES score	45	0.532	0.274–0.718	<0.0002
	More personal electronics owned	44	0.460	0.180–0.671	0.002
	Improved toilet facilities	45	0.406	0.119–0.630	0.006
	Less frequent or no diabetes medications prescribed by their doctor	23	0.503	0.102–0.764	0.014
	Highest IOP	22	0.514	0.117–0.769	0.015

Patients who self-reported higher average glucose levels were also more likely to have high measured blood glucose during the medical mission (r = 0.98, p < 0.001, n = 13). There were no significant associations between IOP and patient-reported barriers to clinic or pharmacy access. This cohort’s elevated IOP may instead be associated with health factors such as drinking water source (p = 0.031), verified through Table 6.

**Table 6:** Partial correlation sensitivity analysis of the association between drinking water source and highest IOP after controlling for diabetes related confounders. To determine whether the association between drinking water source and highest IOP could be explained by diabetes-related factors or nutrition, partial Spearman correlations were run controlling for four covariates individually: prescribed medication use, medication adherence, measured blood glucose, and hemoglobin. The unadjusted correlation between water source and highest IOP was r = 0.51, p = 0.015, n = 22. After controlling for each covariate one at a time, the association remained statistically significant across all four models. Subsample sizes varied due to missing covariate data. The medication adherence model was based on a smaller subsample (n = 10), so a leave-one-out sensitivity analysis confirmed this result was not driven by any single case, with r ranging from 0.73 to 0.91 across all iterations. Highest IOP denotes greatest IOP taken from OS and OD; r = Spearman rank correlation coefficient.

CONFOUNDER CONTROLLED	UNADJUSTED R	UNADJUSTED P	ADJUSTED R	ADJUSTED P	CHANGE IN R	N	INTERPRETATION
Prescribed diabetes medication	0.51	0.015	0.48	0.040	−0.03	20	Water source association to IOP remains significant after controlling for prescribed diabetes medications
Medication adherence	0.51	0.015	0.78	0.013	+0.27	10	Water source association with IOP remain significant after controlling for medication adherence. This was based on a small subsample (n = 10); so a leave-one-out sensitivity analysis confirmed stability (r ranged 0.73–0.91).
Measured blood glucose (mg/dL)	0.51	0.015	0.47	0.043	−0.04	20	Water source association with IOP remains significant after controlling for measured blood glucose.
Hemoglobin levels (g/dL)	0.51	0.015	0.48	0.039	−0.03	20	Water source association with IOP remains significant after controlling for hemoglobin as a proxy for nutrition.

There was no statistical significance between prescribed diabetes medication and IOP in the highest IOP verified dataset (r = −0.36, p = 0.169, n = 16) and should be interpreted with caution until it can be confirmed in a larger study.

### Current use of diabetes medications was associated with higher measured and self-reported blood glucose levels

No patients reported a previous prescription of “*both* tablets and injections” as the survey option accounted for in Table 2.

Instead, patients who were prescribed *either* oral tablets or insulin injections were more likely to be advised that they take their medications daily after meals (r = −0.62, p = 0.003), more likely to have higher measured blood glucose levels (r = 0.55, p = 0.002) and higher self-reported average glucose levels (r = 0.86, p = 0.001). Prescribed patients were also more likely to have seen a doctor recently (r = 0.33, p = 0.038) and less likely to have IOP (r = −0.65, p = 0.007).

### Diabetes burden and effective diabetes management were affected by lifestyle and health-seeking behaviors

Patients whose prescribed therapy included daily medications were more likely to report taking them as prescribed (r = 0.59, p = 0.024), and more likely to have high diabetes burden scores (r = 0.66, p < 0.0006), as graded in Table 2. They were also less likely to exercise weekly (r = 0.47, p = 0.029), and less likely to use private drinking water sources, but instead relied on pipe/tap or shared borehole (r = 0.503, p = 0.014). Patients who reported taking their medications or injections daily were still more likely to have high diabetes burden scores (r = 0.71, p < 0.0007) and a greater number of chronic diseases (r = −0.54, p = 0.027, n = 17). Patients’ symptoms of blurry vision, dizziness, ulcers, or non-healing wounds were associated with high diabetes burden scores (r = 0.56, p < 0.0001). Daily, more frequently occurring symptoms were also strongly correlated with high diabetes burden scores (r = 0.64, p < 0.00003).

There was a strong positive correlation between high diabetes burden scores and: daily medication prescriptions (r = 0.66, p = 0.0006), reported daily adherence (r = 0.71, p = 0.0007), symptom severity and frequency (r = 0.56, p = 0.0001 and r = 0.64, p < 0.00003, respectively), and number of chronic diseases (r = −0.41, p = 0.008). Conversely, the absence of financial difficulty purchasing medications was associated with less severe diabetes burden, and this persisted even after adjusting for SES markers (Table 7).

**Table 7:** Statistical analyses assessing correlations between diabetes burden score and individual barriers to care after adjusting for social determinant risks as confounders.

BARRIER-TO-CARE VARIABLE	β	P-VALUE	INTERPRETATION
Do you have financial difficulty purchasing your diabetes medications?^a^	0.686	0.032*	Having no financial difficulty purchasing medications was associated with a 0.686-unit improvement in diabetes burden score, indicating lower diabetes burden.
Are there people who live with you and take care of you at home?^b^	0.592	0.055^†^	Having in-home caregivers was associated with a 0.592-unit improvement in diabetes burden score, indicating lower diabetes burden, but to be interpreted with caution.
Do you need help to cook, clean, farm/go to the market on your own?	0.338	0.322	No statistically significant association detected in this sample.
Do you smoke?	0.338	0.658	No statistically significant association detected in this sample.
What barriers do you encounter when traveling to clinic/pharmacy?	0.500	0.258	No statistically significant association detected in this sample.
How many times did you drink soda or take sweets in the last week?	0.161	0.798	No statistically significant association detected in this sample.
Use of herbs or natural remedies	0.392	0.491	No statistically significant association detected in this sample.
Frequency of forgetting diabetes medications	−0.470	0.374	No statistically significant association detected in this sample.
Frequency of blood glucose self-monitoring	−0.424	0.519	Not statistically significant. Patients at higher risk may monitor more frequently, which could explain the change in direction.
How often do you exercise?	0.583	0.081^†^	More frequent exercise was associated with a 0.583-unit improvement in diabetes burden score, but did not reach statistical significance.
Time since last doctor visit	0.139	0.447	No statistically significant association detected.
Where do you obtain medication?	−0.568	0.118	No statistically significant association detected. Patients with more severe diabetes burden may use formal/prescribed sources more, which could explain the direction.

^a^ For financial difficulty, “No” = +1 coding; a positive β reflects that those *without* financial difficulty had a more positive diabetes burden score, which means lower burden.

^b^ For in-home caregivers, “Yes” = +1 coding, so a positive β reflects that those *with* caregivers had a more positive diabetes burden score, which means lower burden.

Linear regression models examined the association between each barrier-to-care variable and diabetes burden score after adjusting for type of home, main source of drinking water, and main source of transportation. This analysis tells us which specific barriers to care are associated with worse diabetes burden in this population after removing the influence of socio-economic differences between patients, so that any association found reflects a consistent barrier itself rather than manifestations of the patient’s overall poverty level. Diabetes burden score grading and barriers-to-care response coding are reported in Tables 2 and 3, respectively. *Asterisk indicates statistically significant results (p < 0.05); †Daggers indicate results that approached but did not reach the α = 0.05 threshold. Non-significant results may reflect limited statistical power due to sample size rather than a true absence of association. Note: β denotes the unstandardized regression coefficient.

Only 4 patients self-reported current smoking or living with a family member who smokes, so there were no statistically significant associations between smoking and barriers-to-care score (p = 0.280), diabetes burden score (p = 0.585), blood glucose levels (p = 0.120), or number of chronic diseases (p = 0.791). A comprehensive summary of all significant burdens and barriers related to cataract outcome is provided in Table 8, while modifiable interventions are detailed in Table 9.

**Table 8:** Conclusion table on key findings ranked by significance. This table presents key findings from this study ranked by statistical strength and clinical significance, with the conclusion column addressing their relevance to diabetes burden and cataract outcomes among patients in Imo State, Nigeria. Where multiple associations contribute to a single finding, the strongest p-value was used for ranking. The caregiver finding (Rank 3) did not reach conventional statistical significance for diabetes severity (p = 0.055) but is included given its borderline significance and two additional significant outcomes.

RANK	KEY FINDING	STATISTICAL EVIDENCE	CONCLUSION
1	**Private drinking water source is associated with elevated IOP, independent of medication behavior, glycemic status, hemoglobin levels, and overall SES**.	Highest IOP: r = 0.51, p = 0.015, n = 22. After controlling for medication use r = 0.48, p = 0.040, n = 20; After controlling for adherence r = 0.78, p = 0.013, n = 10; After controlling for blood glucose r = 0.47, p = 0.043, n = 20; After controlling for hemoglobin r = 0.48, p = 0.039, n = 20. After controlling for overall SES score: r = 0.59, p = 0.005, n = 22.	Private drinking water was consistently associated with elevated IOP, making it the most novel finding in this study. The association held after controlling for medication behavior, glycemic status, hemoglobin, and overall SES, showing that water source has an independent association with IOP that cannot be explained by how well or poorly patients manage their diabetes or by general wealth. Water source and blood glucose were not significantly associated with each other (r = 0.13, p = 0.491, n = 32), and blood glucose was not significantly associated with IOP (r = −0.44, p = 0.089, n = 16), suggesting that glycemic status alone doesn’t explain the link between water source and IOP. Therefore, water source may represent a genuinely independent environmental concern for glaucoma risk in this population that warrants further investigation.
2	**Financial difficulty purchasing medications associated with worse diabetes burden.**	β = 0.686, p = 0.032 (adjusted regression).	Financial difficulty was the strongest and only statistically significant predictor of diabetes burden in SES-adjusted analyses. When patients cannot afford medications, their blood glucose stays elevated, which speeds up damage to the blood vessels supplying the lens and retina. This barrier also compounded others; patients with financial difficulty were less likely to have caregivers at home and more likely to need help with daily tasks.
3	**Caregiver presence associated with better diabetes burden, lower barriers to care, and higher hemoglobin simultaneously.**	Diabetes burden: p = 0.055, β = 0.592. Barriers to care: r = 0.327, p = 0.030, n = 36. Hemoglobin: r = 0.463, p = 0.003, n = 36.	Caregiver presence was the only factor in this study linked to better outcomes across all three domains at once. Patients with caregivers had better diabetes management, fewer barriers to care, and healthier Hgb. Each of these pathways connects back to cataract risk since better diabetes management slows cataract lens clouding, fewer barriers means more consistent care, and improved hemoglobin reduces the compounding effect of anemia on blood vessels already damaged by diabetes.
4	**Low hematocrit directly associated with more advanced cataracts.**	Hematocrit and cataract severity: r = −0.742, p = 0.018, n = 12. Hemoglobin and hematocrit: r = 0.952, p < 0.001.	Low hematocrit was directly linked to more advanced cataracts in this sample. When a diabetic patient also has anemia, the blood carries less oxygen to the already compromised vessels supplying the lens and retina. This double burden of diabetic vascular damage and reduced oxygen delivery accelerates lens clouding. Addressing anemia through supplements and locally available nutritional interventions is one of the most modifiable clinical actions in this study.
5	**Need for daily task help associated with advanced cataracts; transportation limitations linked to lower SES and greater struggles farming, cooking, cleaning, and traveling for food**.	Daily task help and cataracts: r = −0.452, p = 0.030. Transport limitation and lower SES: r = 0.371, p = 0.012. Transport limitation and daily task help: r = 0.424, p = 0.004.	Patients who needed help with daily tasks had more advanced cataracts despite having seen a doctor recently. The issue may not be solely primary care access, but access to an eye specialist. Patients with limited transportation were also more likely to struggle with daily tasks, and those daily task limitations were linked to worse cataract outcomes. Bringing specialist eye care closer to the community is the most direct way to address this.
6	**Herb use associated with lower hematocrit (HCT).**	r = −0.473, p = 0.041.	Herb users were more likely to have lower HCT, which is directly linked to worse cataract outcomes in this sample. Herb use likely reflects socio-economic living conditions. Since the use of herbs with metformin and sulfonylureas are not currently monitored in this setting, there is a risk of undetected effects on blood glucose and HCT that should be addressed during doctor visits.
7	**Patients in privately owned homes reported more daily medication use. Patients on more frequent medications had worse diabetes burden, but diabetes burden itself did not differ by socio-economic status, suggesting that medication use reflects disease severity rather than socio-economic advantage**.	Privately owned homes and more daily medication adherence: (r = −0.528, p = 0.017). Prescribed daily medications and worse diabetes burden scores: r = 0.661, p < 0.001, n = 23. Self-reported daily medication adherence and worse diabetes burden scores r = 0.68, p < 0.001, n = 20. Number of chronic conditions and diabetes burden: r = −0.41, p = 0.008, n = 40. Diabetes burden by home ownership group: Non-cement home median = 0 (IQR = 2) vs Owned cement home median = 0 (IQR = 3), p = 0.563. Mann-Whitney U test: p = 0.563 (not significant). Diabetes burden by water source group: Pipe/tap/shared borehole median = −1 (IQR = 2) vs Vendor/bottled/personal borehole median = 0 (IQR = 3), p = 0.899. Mann-Whitney U test: p = 0.899 (not significant). Diabetes burden vs overall SES score: r = −0.07, p = 0.667, n = 45.	Patients in privately owned homes took diabetes medications more consistently than those in rented or shared homes. However, diabetes burden did not differ significantly between the two home ownership groups, nor by water source or overall SES score, meaning higher SES patients were not significantly less burdened by their diabetes. Across the full sample, patients who took medication most frequently and consistently had the worst diabetes burden, not the best, and this pattern held regardless of socio-economic status. So, while better housing was linked to more consistent medication use, it was not linked to less severe diabetes, suggesting that adherence in this sample may simply reflect the need to manage more severe disease rather than leading to better control.

**Table 9:** Modifiable aspects of this healthcare system to improve comorbidity burden and cataract outcomes. The modifiable domains included in this table aim to improve cataract outcomes with respect to diabetes burden, anemia risk, and glaucoma risk among patients in Imo State, southeastern Nigeria. The recommended interventions are based on study findings and tailored to the local healthcare infrastructure and community practices for feasibility. Domains 1 through 5 address the barriers most prevalent among lower SES patients, where access and resource limitations contribute most to diabetes burden. Domains 6 through 9 address the factors most relevant to higher SES patients, where diabetes management behaviors and glaucoma risk are the main concerns.

MODIFIABLE ASPECTS OF THIS HEALTHCARE SYSTEM TO IMPROVE COMORBIDITY BURDEN AND CATARACT OUTCOMES				
MODIFIABLE DOMAIN	STUDY FINDING	RECOMMENDED INTERVENTION	ANTICIPATED BENEFIT	INTERVENTION LEVEL
**Lower SES Patients: Diabetes Burden and Resource Barriers impacting Cataract Outcomes**				
***1. Medication access***				
**Financial difficulty** (Purchasing diabetes medications)	Significant predictor of diabetes severity (β = 0.686, p = 0.032). Also linked to lower caregiver support and more need for daily task help.	Collaborate and support enrolment into the recent 2025 Imo State Health Insurance Agency (IMSHIA) program, which covers metformin and sulfonylureas at a subsidized cost. Follow up with patients between missions to inform them of MIF medication subsidies also available during the year to support continuity of their care.	Better medication access improves adherence, which lowers blood glucose over time. Sustained glycemic control slows lens opacity progression and reduces cataract risk. Addresses the most significant modifiable predictor of diabetes burden found in this study.	Clinical system /Policy/Hospitals and pharmacies
***2. Nutrition and anemia***				
**Limited animal protein intake**; low hemoglobin and hematocrit levels	Low Hgb and HCT were co-associated. HCT associated with more advanced cataracts and greater difficulty with daily tasks. When a diabetic patient also has anemia, the damage diabetes causes to blood vessels gets worse, and the eyes and lens are directly affected.	Crayfish: already used daily in Igbo cooking, and provides heme iron, zinc, and complete protein at very low cost. Moringa (*okwe nri*): grows locally in southeast Nigeria, is high in iron, vitamin C, and folate, and is added to local soups e.g., egusi soup. Cowpeas and African breadfruit (*ukwa*): low glycemic index, high fiber, and affordable. Soaking cowpeas overnight before cooking improves iron absorption. Subsidized protein programs for fish and chicken, local food partnerships, or monthly diabetes-friendly farmer’s market events with incentives for local poultry and fish farmers. Vitamin B12 supplements because plant foods alone cannot provide adequate B12. Low-cost supplements are already co-prescribed at other Nigerian diabetes clinics, so this is feasible to add to routine care visits. Vitamin A: moringa and red palm oil used in moderation are locally accessible and may support eye health.	Treating iron deficiency reduces the burden of diabetic vascular damage and reduced oxygen delivery to the lens and retina. B12 supplements reduce nerve damage that compounds diabetic neuropathy. Low glycemic index food substitutes improve blood sugar management without requiring medication changes. Vitamin A is independently linked to lower cataract risk.	Community/Policy
***3. Caregiver and social support***				
**Absence of in-home** caregiver support	Caregiver presence linked to better diabetes severity score (p = 0.055), lower barriers to care score (r = 0.415, p = 0.005), and higher hemoglobin (r = 0.46, p = 0.003) simultaneously.	Work with local churches and community groups to set up informal caregiver volunteer programs for patients who live alone. Consider paid family caregiver models similar to programs in the United States, where family members are compensated for caring for elderly relatives. This may also create economic opportunity for younger generations with limited formal employment. Train caregivers in medication reminders, help attending clinic appts., and basic dietary guidance during a single mission session. Develop a mobile platform or compile phone resources so caregivers can access guidance on minor issues remotely to reduce unnecessary travel. Assign community health workers to patients with no caregiver, low SES, and limited transportation.	Caregiver support is the only finding in this study associated with better diabetes outcomes, lower barriers, and better hemoglobin levels at the same time. Better medication management and nutrition together lower diabetes severity, which directly improves cataract surgery access and outcomes. Paid caregiver models may create economic opportunity while sustaining patient support. Remote access tools reduce transportation burden for minor concerns between missions.	Community/System/Policy
***4. Transportation and healthcare access***				
**Limited transportation** options	Patients with transportation limitations had advanced cataracts despite recent doctor visits, suggesting the issue may be access to an eye care specialist rather than primary care.	Use a mobile clinic model to bring diabetes screening and medication dispensing directly to the community. Schedule clinic days to match local market days when transport is naturally more available or conduct weekly screenings after church-service Offer transport vouchers or coordinate with local churches to provide rides on clinic days. Introduce medication delivery as an alternative to pharmacy pickup for patients with severe mobility limitations. Prioritize home visits by community health workers for patients with advanced cataracts who cannot travel independently.	Bringing care to the community increases clinic attendance and medication refill rates. Earlier cataract care leads to better surgical outcomes and preserved vision.	System Community
***5. Culturally sensitive clinical care***				
**Regulated herb use**; anemia screening	Patients managing their diabetes with “onugbu” were more likely to have lower hematocrit. Herb and drug interactions are not currently monitored in this setting.	Ask about specific and detailed herb use at every diabetes visit. Counsel patients on common herb interactions with metformin and sulfonylureas. Train local pharmacists to provide accessible guidance on safe herbal remedy use alongside prescribed medications. Add hemoglobin or hematocrit testing to routine diabetes care visits using a point-of-care device. Track missed doctor visits among patients with dietary limitations using simple community health worker checklists and follow up with home visits for high-risk patients.	Finding low hematocrit early means anemia can be caught and treated before it starts affecting vision or nerve function. Teaching patients about herb interactions helps prevent hidden effects on blood sugar and blood cell levels that no one would otherwise know about.	Clinical system
***6. Higher SES patients: glaucoma risk and management of comorbidities impacting cataract outcomes***				
**Private drinking water source** associated with elevated IOP	Private water access associated with higher SES yet higher IOP in both eyes (r = 0.51, p = 0.015). Novel finding suggesting environmental exposure may contribute to glaucoma risk beyond traditional clinical factors.	Introduce routine IOP screening as part of diabetes care visits for higher SES patients. Glaucoma risk is disproportionately high in West African populations and is often asymptomatic until advanced. Test private borehole water sources for specific mineral content and potential contaminants using low-cost community water testing kits. Recommend low-cost household filtration options for patients using private borehole sources. Develop community education on water safety and its potential relationship to eye health, in partnership with local environmental monitoring programs.	Routine IOP monitoring enables earlier detection of glaucoma risk before vision loss occurs. Identifying environmental contributors to elevated IOP opens a novel prevention pathway specific to this population. Water safety education addresses a modifiable exposure that may affect outcomes beyond eye health.	Community/Policy
***7. Exercise and lifestyle***				
**Low exercise frequency** among medicated patients	Higher SES patients prescribed daily medications were less likely to exercise weekly (r = 0.47, p = 0.029), suggesting medication alone is not translating into healthier behaviors.	Incorporate structured physical activity counseling into diabetes care visits. Recommend culturally familiar activities such as farming, walking, or dancing that are already part of daily life. Provide simple written or illustrated exercise tips that patients can take home and share with their caregivers. Use community health workers to follow up on lifestyle goals like exercise frequency during visits.	Regular exercise improves insulin sensitivity and lowers blood glucose independently of medication. Physical activity reduces cardiovascular risk factors that compound diabetic eye disease. Addressing sedentary behavior in medicated patients may gain diabetes improvements that medication alone is not achieving.	Clinical/Community
***8. Diabetes management quality***				
**High diabetes severity** despite daily medication use	Patients on medications still had high diabetes burden scores, more chronic diseases, and frequent symptoms including blurry vision, dizziness, ulcers, and non-healing wounds (r = 0.64, p < 0.00003).	Review medication regimens for patients with high diabetes severity scores despite reported adherence. Consider whether their current first-line agents are adequate or need escalation. Introduce structured symptom tracking at every visit. Blurry vision, dizziness, ulcers, and non-healing wounds should be documented as indicators of disease progression. Coordinate referrals to specialist care for patients with multiple chronic diseases and persistent symptoms. Provide patients with simple “symptom diaries” or mobile-based tools to track daily symptoms between visits.	Identifying patients whose disease is progressing despite medication allows us to review their regimen on time before complications Tracking blurry vision systematically creates a direct link to timely eye care referral.	Clinical system
***9. Blood glucose self-monitoring***				
**Infrequent blood glucose self-monitoring**	81% of patients (n = 29/36) reported monitoring less than once per week. No significant association was found between monitoring frequency and IOP, r = −0.25, p = 0.297, or any other clinical variable in this dataset.	Provide affordable glucometers and test strips to patients who lack home monitoring equipment. Educate patients on the importance of regular blood glucose self-monitoring as part of standard diabetes self-management.	More frequent monitoring allows patients to detect and respond to glycemic fluctuations.	Clinical/Community

## Discussion

This study identified several significant socio-economic, clinical, and behavioral factors associated with poor diabetes management and comorbidity risks in cataract patients in rural Nigeria. Many patients had high diabetes burden scores despite being prescribed daily medications and reporting adherence to those medications, suggesting that there are more complex underlying barriers to effective disease management. Barriers such as financial difficulty, limited access to care, and reliance on herbal remedies were prominent. Other comorbidity risks like anemia and glaucoma were also variables associated with diabetes care, cataract severity, and surgery access. Elevated IOP, a risk factor for glaucoma, was associated with private drinking-water sources, while anemia risk appeared connected to both financial difficulty and herbal medicine use.

Our findings reveal personal and socio-economic barriers to diabetes care that may delay or preclude cataract surgery access. Medication adherence alone was not a reliable measure of diabetes control since many adherent patients still had higher glycemic scores. This likely reflects more severe disease before diagnosis or adherence began, especially since frequent symptoms and more chronic diseases were significantly tied to high diabetes burden scores.

Most patients do not check their blood glucose weekly, no patients check daily, and among the small subset who monitored their glucose weekly, they were more likely to have seen a doctor recently. The limited monitoring may reflect the lack of access to a personal testing device or a lack of awareness about the importance of frequent self-monitoring. Many patients may be relying on their next doctor visit to get updates on their diabetes.

Diabetes care measures, such as frequent checks, are important since they were associated with reduced IOP in cataract patients, possibly a reduced risk of glaucoma (a prevalent comorbidity in our cohort that could interfere with their surgery access). Glaucoma currently has no complete cure, and vision loss cannot be reversed. It can only be managed with daily medications, eye drops, or surgery to slow progress and reduce the risk of vision loss, if detected early [19]. Therefore, it is important to understand the association between private drinking water sources and elevated IOP before it progresses into irreversible blindness.

Approximately 38.1% of 42 patients reported using herbs and traditional remedies such as ointments for leg pain, pepper leaves, alomo bitters, and onugbu (*Vernonia amygdalina* or bitter leaf) to manage their diabetes and reduce the burden of their symptoms. A few also reported the use of blood tonics. The observed use of *V. amygdalina* aligns with existing research on its biological potential against several diseases, including diabetes, where the juice extracted from the leaf, often mixed with salt, is traditionally taken for diabetes treatment in Nigeria [20–22]. Many patients reported using the leaf in this way during interviews, although no one mentioned the addition of salt. Herb use was especially common among patients living in more crowded homes, suggesting that socio-economic factors and shared cultural knowledge may be influencing the reliance on herbal remedies. Second, herb users in our study were less likely to consume sugary foods and sodas multiple times per week, which could reflect either more intentional efforts to manage their diabetes or simply limited access to such foods. Our study showed no significant association between herb use and blood glucose levels. However, there was a significant association between herb use and lower HCT levels, raising concerns about anemia risk. Lower HCT was also significantly associated with more severe cataracts. If behaviors aimed at managing their diabetes are instead associated with systemic risks such as anemia, which may still further limit patients’ eligibility for cataract surgery, then detailed education and clinical screening become essential to reduce unintended harm and improve surgery access. *V. amygdalina* has been recognized for anemia risk in prolonged or high-dose use, but also for antioxidant benefits when taken in specific appropriate doses, and its supportive role in managing human sickle cell disease in vitro [23–26]. These all suggest that the health effects of the herb may vary depending on the preparation method, dosage, and duration of use [23, 27–29]. In this rural setting, where patients face transportation and financial barriers and where patients with high barriers to care are less likely to receive their medications from a pharmacy with a prescription, community education is essential. This education should respect traditional practices while addressing potential risks and offering guidance on safer herb preparation and appropriate, individualized dosing. More research is needed to better understand the long-term hematologic impacts of herbal use in diabetes care.

Our results support existing research showing that SDOH, such as transportation, food security, housing, and caregiver support, have measurable impacts on chronic disease outcomes [30–32]. Caregiver presence may improve future eligibility for cataract surgery in two key ways. First, by assisting patients with advanced cataracts who reported reduced independence and a need for help with daily activities such as cooking, cleaning, farming, or going to the market. Second, by helping reduce the burden of comorbidities like anemia, since caregiver support was significantly associated with higher Hgb and HCT levels in our study cohort. Patients with lower HCT were more likely to need help accessing and preparing nutrient-rich foods necessary for maintaining healthy HCT levels, particularly foods high in iron and protein [33, 34]. Since low HCT was also strongly associated with more advanced cataracts (Figure 2), minimizing this extra burden of low HCT is especially important. A 2021 MIF study done in the same community suggests that caregiver support could improve access to a balanced diet. This may, in turn, improve HCT levels. Similarly, patients who could not easily travel to the clinic/pharmacy due to transportation or physical disability had a strong association with their diabetes affecting what they could eat. Many described experiencing pain or discomfort, such as feeling like “stones inside me [them],” whenever they consumed common staple foods, specifically yam, rice, apples, or fried items. Despite this, they remained heavily reliant on charity food sources, indicating that both food insecurity and physical limitations are barriers to their diet. Among those whose diabetes limited what they could eat, they also had significantly higher barriers and were less likely to have seen a doctor recently. These finding suggest that access to care, food choice limitations, and a lack of caregiver support systems influence the barriers to care and the severity of the systemic diseases seen in the community. The SDOH barriers in this rural community report similar findings to community studies in the United States that link improved food access and transportation to lower blood pressure and improved diabetes outcomes [35–39]. Financial difficulty purchasing medications and the absence of in-home caregiver support were the most significant modifiable predictors of diabetes severity in this sample, even after accounting for socio-economic differences between patients (Table 7). Barriers that did not reach statistical significance after SES adjustment, such as smoking, should not be interpreted as unimportant; rather, in this subsample of patients, they were not what was driving the differences in diabetes severity between patients, and a larger sample may reveal true effects. Taken together, these findings suggest that helping patients afford their medications and strengthening social support in the home may offer the greatest opportunity to reduce diabetes burden and improve cataract outcomes in this community.

We examined the link between glaucoma risk and drinking water source in our study, as presented in Figure 3. Currently, no studies directly examine whether the type or source of drinking water, such as borehole, vendor (sachet), or bottled, affects IOP. Most existing literature focuses on the physiological response to water volume and the timing of intake, rather than its composition or origin. The association between drinking water source and highest IOP persisted after controlling for potential confounders including medication behavior, glycemic status, hemoglobin (Table 6), and overall SES, suggesting that it is not mediated through diabetes management or SES. Neither Hgb nor HCT (our nutrition markers) was significantly associated with water source or IOP, which ruled out the hypothesis of a nutrition-anemia pathway impact on IOP.

The mechanism behind this IOP–water source association remains unexplained by the clinical and socio-economic variables available in our dataset. The fact that the association held up after individually controlling for all our key clinical and socio-economic variables with sufficient sample size (medication behavior, glycemic status, hemoglobin, and overall SES) suggests that something specific to the water itself, rather than who is drinking it, may be driving this IOP–water source relationship. Prior research in this community has linked private water source to hypertension risk [1], suggesting that water source may represent a broader environmental risk factor for chronic disease in this population beyond diabetes management alone.

We hypothesize that mineral content or contaminants in private water sources could influence systemic health in ways that could affect ocular perfusion and IOP. In 2021, Nzenwa et al. appraised the boreholes in the Owerri municipal community in Imo State and found that they met WHO’s Water Quality Index (WQI) standards for safe consumption, with a WQI of 24.91 to 70.06, except for turbidity in specific locations within the community. However, the WHO Guidelines for Drinking-Water Quality (GDWQ) are necessary to accurately define borehole water as a potable water source [40]. The GDWQ is different in that it tests for additional parameters such as lead, arsenic, fluoride, and specific pesticides, which have been linked to an increased risk of glaucoma in several studies [40–43]. The proposed mechanisms include neurotoxicity, inflammation, and increased oxidative stress, which can damage the optic nerve.

A second hypothesis could be an association between hypertension’s vascular stress and elevated IOP, since the 2018 MIF study by Anderson et al. in the same Imo State community similarly found a significant association between personal borehole use and hypertension [1]. Multiple studies confirm a clear, direct correlation between hypertension and IOP. In a large Japanese cross-sectional study, every 10 mmHg increase in SBP was associated with a 0.32 mmHg rise in IOP, and every 10 mmHg increase in diastolic pressure corresponded to a 0.41 mmHg increase, even after adjusting for age, sex, body mass index, diabetes status, and antihypertensive use [44]. Participants with hypertension had an average IOP of 14.4 mmHg, compared to 13.7 mmHg in those without hypertension. Meta-analyses in other studies further supported a modest but positive relationship between SBP increases and IOP, including a 1.69 risk ratio of developing open-angle glaucoma (OAG) in hypertensive patients [45]. While these increases are consistent, they do not fully explain the much higher IOP levels (>21 mmHg) observed among patients who drink from private water sources in our study. Importantly, evidence from an animal study suggests that the duration of hypertension may be critical. Chronic hypertension sustained over four weeks impaired ocular perfusion and increased susceptibility to IOP elevation, whereas acute hypertension did not have the same effect [46].

Similarly, chronic direct exposure to unregulated parameters may contribute to the higher prevalence of elevated IOP observed in patients with private boreholes (83.33%) compared with those using shared boreholes (27.27%). It is plausible that individuals relying exclusively on private boreholes consume larger volumes of water from a single, unregulated source, thereby increasing their cumulative exposure to potentially harmful compounds. In contrast, shared boreholes distribute consumption across multiple households, which may lower individual exposure risk. These hypotheses warrant further investigation.

The patterns in disease risk from borehole sources raise concerns about the safety and mineral content of private drinking water in the community. In the context of our findings, where higher IOP was strongly associated with private water source, further research is needed to explore whether vascular stress with chronic hypertension, or simply community-specific environmental exposures, may be contributing to glaucoma risk in this population. Our study cannot infer causality; it echoes global findings suggesting that drinking-water quality and accessibility may shape systemic health.

Consistent water testing in private homes and a deeper understanding of groundwater drilling depth and aquifer conditions in the local community should be evaluated to determine whether the boreholes are vulnerable to nitrogen runoff or seasonal storm surges, as the Anderson et al. study raises as a concern in other areas.

## Limitation

This study is limited by its small sample size and cross-sectional design, which restricts causal interpretations. The self-reporting nature of our cross-sectional study may also be subject to patient recall bias. Nonetheless, our modified MIF SES questionnaire tested for diverse socio-economic variables ranging from the type of toilet facilities in the home to meat consumption, and even details on the drinking-water source. This allowed for a unique analysis of the impact of these often-overlooked associations with health-seeking behaviors and comorbidity severity. The identified barriers in this rural community can be adapted into other community-based research seeking to effectively utilize medical missions to provide health stability, sustainable solutions, and improve health outcomes in other rural communities. Future research should include cohort tracking of blood glucose and cataract progression, an evaluation of culturally sensitive interventions (e.g., education on regulated herbal use), and an assessment of water quality’s impact on glaucoma and hypertension. Expanding access to affordable glucometers and introducing community-based diabetes programs may help improve self-monitoring and support more effective diabetes management. These steps could enhance patient readiness for cataract surgery during future medical missions and leave the community better equipped to identify systemic disease risks, manage chronic illnesses, and maintain their health and independence even outside of medical mission periods.

## Conclusion

Diabetes management among cataract patients in low-resource settings is impacted by an interplay of socio-economic and systemic health barriers summarized in Table 8. Effective interventions must go beyond prescription access to address the root determinants of comorbidities since some comorbidities appear more likely in either high SES or low SES groups. Socio-economic factors, such as food insecurity, limited transportation, environmental health hazards, health management education, and culturally grounded healthcare behaviors, detailed in Table 9, are specific targets for mitigation in the community. If successfully addressed, the prevalence and severity of comorbidities interfering with cataract surgery among this cohort may reduce, barriers to treatment may reduce, participants’ knowledge of effective and personalized self-management may improve, and cataract surgery may become more accessible and feasible in future medical missions.

## Data Access Declaration

All authors had access to the data and participated in the manuscript and research.
